# Copper-Induced Enhancement of Glioblastoma Tumorigenicity via Cytochrome C Oxidase

**DOI:** 10.3390/antiox14020142

**Published:** 2025-01-24

**Authors:** Claudia R. Oliva, Md Yousuf Ali, Susanne Flor, Corinne E. Griguer

**Affiliations:** 1 Free Radical & Radiation Biology Program, Department of Radiation Oncology, University of Iowa, Iowa, IA 52242, USA; claudia-oliva@uiowa.edu (C.R.O.); mali1@mgh.harvard.edu (M.Y.A.); susanne-flor@uiowa.edu (S.F.); 2 Mass General Hospital Center for Cancer Research, Harvard Medical School, Boston, MA 02129, USA

**Keywords:** copper, oxphos, glioblastoma, cytochrome c oxidase

## Abstract

Copper is an essential trace element, yet chronic copper exposure can lead to toxicity in humans, and high levels of copper have been found in the blood or tumors of patients with various forms of cancer and may affect cancer severity and response to treatment. Copper is required for the activation of cytochrome c oxidase (CcO), the mitochondrial complex that facilitates oxidative phosphorylation (OXPHOS)-mediated ATP production. We recently reported that the increased activation of CcO underlies the acquisition of treatment resistance in glioblastoma (GBM) cells. However, the potential role of copper in GBM progression or treatment resistance has not been investigated. Here, we present evidence that exposure to 20 µM copper, the maximum allowable limit for public water supplies set by the U.S. Environmental Protection Agency, promotes GBM tumor growth and reduces overall survival in vivo and increases GBM cell resistance to radiation and chemotherapy in vitro. In vitro exposure to 20 µM copper substantially increased the activity of CcO, elevated the rate and level of ATP production, and triggered a metabolic shift to an OXPHOS phenotype in GBM cells. Furthermore, copper exposure led to a substantial increase in the accumulation of glutathione and glutathione precursors in these cells. These findings establish copper as a tumor promoter in GBM and suggest that copper mediates these effects through the upregulation of CcO activity, which enhances OXPHOS metabolism and glutathione production.

## 1. Introduction

Copper is an essential micronutrient for human development and health. As a critical catalytic cofactor or structural component in various proteins, copper plays key roles in vital biological processes, including enzyme activity, oxygen transport, and cell signaling [1]. Intracellular copper levels are tightly controlled, and genetic mutations or environmental factors that alter copper homeostasis can cause a range of pathophysiologic effects in humans [2,3]. Chronic copper exposure is known to induce liver, renal, and central nervous system toxicity in humans [4,5,6,7]. In addition, elevated levels of copper have been detected in the serum and/or tumors of patients with certain types of cancer, including breast [3,8,9,10,11,12], lung [13,14,15], gastrointestinal [16,17,18,19,20], oral [21], thyroid [22], gallbladder [23], gynecologic [16,24], prostate [25], and brain cancer [26,27,28,29,30,31], and these high copper levels often correlated with cancer severity and response to therapy [32,33]. Additionally, in vitro and in vivo studies indicate that copper may be rate limiting for tumor growth [34,35,36]. However, the mechanisms by which intracellular copper promotes tumor progression remain incompletely defined.

Copper is redox active, readily transitioning between Cu⁺ and Cu^2^⁺ states as it donates and accepts electrons and thereby facilitates numerous biological redox reactions. In prokaryotes, more than 10 copper-dependent proteins, including cytochrome c oxidase (CcO), have been identified. CcO serves as the terminal enzyme in the respiratory chain across all eukaryotes [37,38]. In this capacity, CcO activity is required for oxidative phosphorylation (OXPHOS) to occur, as it links the redox transport of electrons through the respiratory chain to proton translocation and aerobic ATP production [39,40]. Notably, the catalytic center of CcO contains two copper sites, CuA and CuB, which collectively accommodate three copper ions that are required for the assembly, stability, and enzymatic activity of CcO [39]. Recent preclinical studies in the animal models of pancreatic cancer and clear cell renal cell carcinoma have suggested that the upregulation of CcO activity, and thereby OXPHOS, in the tumor cells largely underlies the tumor-promoting effects of excess copper [34].

Our previous studies have shown that elevated CcO activity and the corresponding metabolic switch to OXPHOS are linked to chemoresistance and radioresistance in glioblastoma (GBM), the most aggressive and common type of primary brain tumor [41,42,43,44]. Although research published in the 1990s revealed the therapeutic benefits of systemic copper depletion in animals implanted with VX2 carcinoma or experimental 9L-gliosarcoma [45,46] as models of brain cancer, whether excess copper mediates the upregulation of CcO activity to facilitate GBM progression remains unknown. In this study, we tested the hypothesis that bioavailable copper enhances tumorigenicity and treatment resistance in GBM via the activation of CcO.

## 2. Materials and Methods

### 2.1. Cell Culture

Established human malignant glioblastoma multiforme cell lines (U251, U251-deltaCOX4 clones #3 and #4, patient-derived JX22, JX39, JX59, and D456) [41,42,44] were grown in DMEM/F-12 medium, supplemented with 7% heat-inactivated FBS and L-glutamine and incubated at 37 °C in a humidified atmosphere containing 5% CO_2_. Normal human astrocytes (ScienceCell Research Laboratories, Carlsbad, CA, USA) were isolated from human brain (cerebral cortex) and cryopreserved at passage one. NHAs were cultured in astrocyte medium (ScienceCell Research Laboratories, catalog # 1801). Cells were treated with CuSO_4_ (Sigma-Aldrich, St. Louis, MO, USA) and/or tetrathiomolybdate (Sigma) at the concentrations and durations specified in the figures. Cultures were regularly tested for mycoplasma contamination and authenticated by short tandem repeat (STR) profiling.

### 2.2. Enzymatic Activities

Activity levels of citrate synthase (CS) and CcO were determined in isolated mitochondrial fractions as we previously described [44,47].

### 2.3. Measurement of Radioactive Copper Uptake in Cells

To assess the uptake of radioactive copper, cells were treated with 18.5 kBq of ^64^CuCl_2_ (University of Wisconsin Madison, Department of Radiology). At 0.5, 1, and 2 h after the addition of ^64^CuCl_2_, cells were removed from the incubator, medium was removed, and cells were washed three times with ice-cold PBS. Cells were then lysed using 1 mL of 0.5 M NaOH and transferred into 10 mL of Econo-SafeTM liquid scintillation cocktail (Research Product International, Mount Prospect, IL, USA). Radioactivity was counted for 2 min on a Tri-Carb 2800TR Liquid Scintillation Analyzer (PerkinElmer, Waltham, MA, USA). ^64^Cu uptake was expressed as the percentage of the initial radioactivity dose normalized to the amount of protein.

### 2.4. High-Energy Redox Metabolomic Analysis

After 48 h of treatment with or without 20 µM copper, cell culture plates were washed twice with ice-cold PBS followed by two washes with ice-cold water and then flash-frozen using liquid nitrogen. Cell culture plates were lyophilized overnight and then scraped into 1 mL of ice-cold 2:2:1 methanol/acetonitrile/water containing a mixture of internal standards to extract metabolites. The mixtures were transferred to a microcentrifuge tube and flash-frozen in liquid nitrogen, thawed for 10 min in a water bath sonicator, and rotated for 1 h at −20 °C. Mixtures were then centrifuged for 10 min at 21,000× *g*, and 300 µL of the cleared metabolite extracts were transferred to autosampler vials and dried using a SpeedVac vacuum concentrator (Thermo Fisher Scientific, Carlsbad, CA, USA).

Liquid chromatography–mass spectrometry: Dried extracts were reconstituted in 25 µL acetonitrile/water (1:1 *v*/*v*), vortexed well, incubated at −20 °C overnight, and centrifuged the next day. The supernatant was then transferred to liquid chromatography–mass spectrometry (LC-MS) autosampler vials for analysis. LC-MS data were acquired on a Thermo Scientific Q Exactive Hybrid Quadrupole Orbitrap mass spectrometer with a Vanquish Flex UHPLC system or Vanquish Horizon UHPLC system.

The LC column used was a Millipore SeQuant ZIC-pHILIC (2.1 × 150 mm, 5 µm particle size) with a ZIC-pHILIC guard column (20 × 2.1 mm), with an injection volume of 2 µL. The mobile phase was as follows: Solvent A: 20 mM ammonium carbonate [(NH_4_)2CO_3_] and 0.1% ammonium hydroxide (*v*/*v*) [NH4OH], pH ~ 9.1; Solvent B: acetonitrile. The column was run at a flow rate of 0.150 mL/min.

The mass spectrometer was operated in full-scan, polarity-switching mode from 1 to 20 min, with the spray voltage set to 3.0 kV, the heated capillary held at 275 °C, and the HESI probe held at 350 °C. The sheath gas flow was set to 40 units, the auxiliary gas flow was set to 15 units, and the sweep gas flow was set to 1 unit. MS data acquisition was performed in a range of *m*/*z* 70–1000, with the resolution set at 70,000, the AGC target at 1 × 10^6^, and the maximum injection time at 200 ms.

Acquired LC-MS data were processed by Thermo Scientific TraceFinder 4.1 software, and metabolites were identified based on the University of Iowa Metabolomics Core facility standard-confirmed, in-house library. NOREVA was used for signal drift correction. Data were normalized to the sum of all the measured metabolite ions in that sample.

### 2.5. ROS Levels

Cells were treated with the indicated concentrations of copper for specified durations. Additional groups were treated with 2 mM L-buthionine-S,R-sulfoximine (BSO) for 48 h to deplete glutathione or 10 µM tetrathiomolybdate (TM) for 48 h to chelate copper. After treatment, 4 × 10⁴ cells/well were seeded into black 96-well plates with clear flat bottoms. After 24 h, cells were exposed to H^2^O^2^ as per the experimental design. ROS levels were quantified 30 min post-treatment using the Cellular Reactive Oxygen Species Detection Assay Kit (Abcam, Waltham, MA, USA. Cat No. ab186027), following the manufacturer’s protocol. Fluorescence (λ ex/em 520/605 nm) was measured using a Tecan Infinite 200 microplate spectrofluorometer (Morrisville, NC, USA).

### 2.6. Xenograft Mouse Model with Intracranial Tumors

All animal experiments and surgical procedures were conducted with approval from the Institutional Animal Care and Use Committee of the University of Iowa. Six-week-old female athymic nude mice were obtained from Envigo (Indianapolis, IN, USA) and randomly divided into four groups of nine. Two groups received treatment with 20 µM CuSO_4_ in drinking water for 7 days prior to intracranial tumor establishment, with treatment continuing thereafter. A total of 3 × 10^5^ U251 (18 mice) or Jx22 (18 mice) patient-derived human glioma cells, suspended in 5% methylcellulose in serum-free medium, were injected intracranially as previously described [42,48]. The primary endpoint of this study was animal survival; moribund animals that became unresponsive to mild external stimuli were euthanized, and this date was recorded as the estimated date of death. Brains were harvested, fixed in 10% neutral buffered formalin, routinely processed, and embedded in paraffin. Tissue sections were cut at 5 µm and stained with hematoxylin and eosin (H&E) for histological examination.

### 2.7. Clonogenic Assays

Patient-derived U251 and Jx22 cells were pretreated with 20 µM CuSO_4_ for 72 h either with or without 10 µM TM and irradiated with doses of 2–8 Gy at a dose rate of 0.65 Gy/min^−1^ using a 6000 Ci^137^Cs source. Clonogenic survival fractions were determined after irradiation, as we previously described [41,49]. Colonies were then fixed, stained, and counted in a blinded fashion with a colony counter pen. Plating efficiency (PE) was considered to be the ratio of the number of colonies to the number of cells seeded, and the clonogenic survival fraction (SF) was determined based on the number of clones surviving treatments, correlated to the PE of non-treated control cells. Equation used: PE = (colonies counted/cells plated) × 100, and SF = (PE of treated sample/PE of control) × 100.

### 2.8. Crystal Violet Proliferation Assay

Cell survival determination was performed as we previously described [48]. Briefly, after treatment, cells were fixed with ice-cold 3.7% paraformaldehyde, washed with PBS, and stored at −20 °C overnight. Cells were then stained with 0.05% crystal violet solution and dried, then the dye was solubilized with 10% acetic acid, and absorbance was measured at 590 nm.

### 2.9. Determination of Apoptosis

The apoptosis-inducing effect of temozolomide (TMZ) was determined by flow cytometry using an annexin-V/propidium iodide (PI) assay as we described previously [42,49]. About 1 × 10^5^ cells were plated in each well of a 12-well plate and treated either with vehicle or 300 or 500 µM TMZ with and without 20 µM CuSO_4_ for 48 h. Apoptosis was determined using the PE Annexin V Apoptosis Detection Kit (Becton, Dickinson, San Diego, CA, USA), according to the manufacturer’s instructions, and analyzed by flow cytometry using a Becton Dickinson LSR II flow cytometer using 488 nm excitation and 530 nm emission wavelengths. The median fluorescence intensity of 10,000 cells was determined with FlowJo Software Version 10 (Becton, Dickinson).

### 2.10. Statistics

All data were evaluated using GraphPad Prism (GraphPad Software, version 10.4.1, San Diego, CA, USA). Results are expressed as the mean ± SEM, and *p* < 0.05 was considered significant. Statistical analyses were performed using one-way analysis of variance (ANOVA), followed by Tukey’s multiple comparison test or (un)paired Student’s *t* test. Statistical significance was indicated with asterisks: * *p* < 0.05, ** *p* < 0.01, *** *p* < 0.001, and **** *p* < 0.0001.

## 3. Results

### 3.1. Bioavailable Copper Increases CcO Activity in GBM Cells

**Figure 1:**
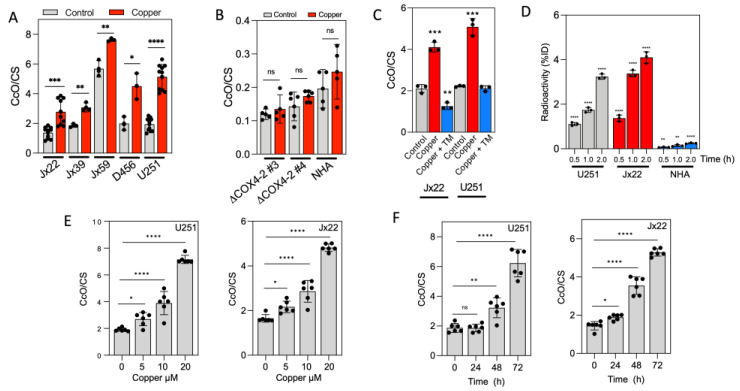
Excess copper increases CcO activity in GBM cells. (**A**) CcO activity in patient-derived GBM cells treated with vehicle (water) or 20 µM copper for 48 h. (**B**) CcO activity in U251-deltaCOX4 clones and NHAs treated with 20 µM copper for 48 h. (**C**) CcO activity in U251 and JX22 cells treated with 20 µM copper in the presence or absence of TM (10 µM). (**D**) ^64^Cu uptake over time in U251, JX22, and NHA cells. (**E**) Dose-dependent effects of copper on CcO activity in U251 (left), JX22 (right) cells. (**F**) Time-dependent effects of copper (20 µM) on CcO activity in U251 (left) and JX22 (right) cells. Data are presented as the mean ± SEM. * *p* < 0.05, ** *p* < 0.01, *** *p* < 0.001, and **** *p* < 0.0001, calculated by Student’s *t* test. CS, citrate synthase; ns, not significant.

In a previous study by Ishida et al. [34], bioavailable copper promoted pancreatic tumor progression in mice, even at the upper concentration limit (20 µM) allowed in public drinking water by the Environmental Protection Agency (EPA). Therefore, we investigated the impact of 20 μM bioavailable copper on CcO activity in GBM cells in vitro. Exposure to 20 μM copper for 72 h significantly increased CcO activity in all glioma cell lines tested (Figure 1A). The depletion of CcO subunit 4 (COX4), which prevents CcO activation [42], abolished the stimulatory effect of copper on CcO activity. Importantly, exposure to copper did not induce significant changes in CcO activity in normal human astrocytes (NHAs), suggesting a selective response to copper by cancer cells (Figure 1B). A further analysis focused on the two glioblastoma cell lines that exhibited the most significant increases in CcO activity following copper treatment: JX22 and U251. Treatment with the copper chelator TM (10 μM) completely abrogated the stimulatory effect of copper on CcO activity, reducing CcO activity by threefold in both JX22 and U251 cells (Figure 1C).

Next, we assessed the cellular uptake of ^64^Cu in U251 and JX22 glioma cells as well as in NHAs. ^64^Cu uptake was expressed as the percentage of initial radioactivity dose normalized to the amount of protein. The cellular uptake of ^64^Cu increased as a function of incubation time in each cell line, but the uptake by tumor cell lines far exceeded the uptake by NHAs (Figure 1D). To explore the dose dependency of copper treatment, we incubated U251 and JX22 cells with increasing doses of copper (0, 5, 10, and 20 μM) for 72 h. In both cell lines, treatment with 5 μM copper elicited a minimal but significant increase in CcO activity, whereas treatment with 10 μM and 20 μM elicited far more substantial increases in CcO activity of approximately 2-fold and 4-fold, respectively (Figure 1E). In time-course experiments, exposure to copper for 24 h increased CcO slightly but significantly in JX22 cells but did not affect CcO activity in U251 cells. However, exposure to copper for 48 or 72 h substantially increased CcO activity in both cell lines (Figure 1F).

**Figure 2:**
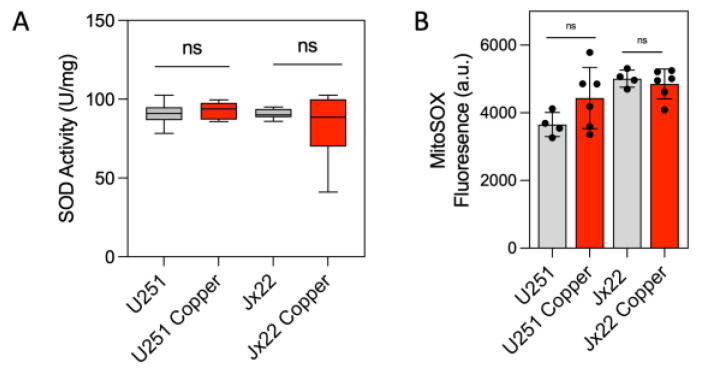
Excess copper does not affect SOD activity in GBM cells. (**A**) SOD activity in U251 and JX22 cells treated with vehicle (water) or 20 µM copper for 48 h. (**B**) Quantification of mitochondrial O_2_^•−^ production in U251 and JX22 cells treated with vehicle or 20 µM copper for 48 h. Data are presented as the mean ± SEM (n = 6). ns, not significant, calculated by Student’s *t* test.

To assess whether the observed copper-induced effects were specific to CcO or extended to other copper-containing enzymes, we examined the activity of superoxide dismutase (SOD). Interestingly, treatment with copper did not affect SOD activity (Figure 2A). Accordingly, exposure to 20 μM copper for 72 h did not significantly affect superoxide production in either cell line (Figure 2B).

### 3.2. Bioavailable Copper Rewires GBM Metabolism

**Figure 3:**
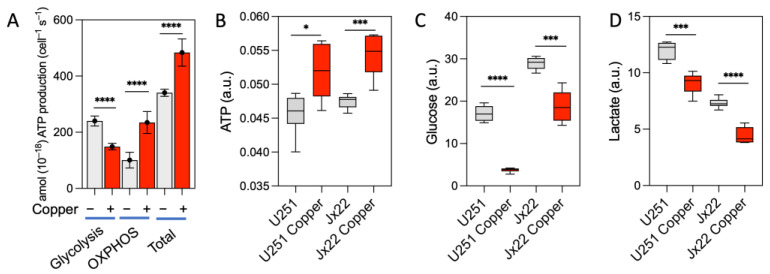
Excess copper promotes an OXPHOS phenotype in GBM cells. (**A**) ATP production rate in U251 cells treated with vehicle or 20 µM copper for 48 h. (**B**) Steady-state levels of intracellular ATP in U251 and JX22 cells treated with vehicle or 20 µM copper for 48 h. (**C**,**D**) Quantification of cellular glucose (**C**) and lactate (**D**), estimated by GC-MS, in U251 and JX22 cells treated with vehicle or 20 µM copper for 48 h. Data are presented as the mean ± SEM (n = 6). * *p* < 0.05, *** *p* < 0.001, and **** *p* < 0.0001, calculated by Student’s *t* test.

We next assessed the metabolic phenotype of control and copper-exposed cells, determining the ATP production rate and the extent of cellular glucose uptake and lactate production to reflect glycolytic activity. Exposure to copper caused a significant reduction in the rate of glycolysis-driven ATP production that was accompanied by an increase in the rate of OXPHOS-driven ATP production, ultimately increasing the overall ATP production rate (Figure 3A).

In control U251 cells, the rate of ATP production from glycolysis alone was 240 ± 18 amol cell^−1^ s^−1^, and the rate of mitochondrial ATP production was 100 ± 28 amol cell^−1^ s^−1^, yielding a total ATP production rate of 340 ± 13 amol cell^−1^ s^−1^. However, in the copper-treated U251 cells, the rate of ATP production from glycolysis was 148 ± 12 amol cell^−1^ s^−1^ and the rate of mitochondrial ATP production was 235 ± 39 amol cell^−1^ s^−1^, yielding a total ATP production rate of 484 ± 49 amol cell^−1^ s^−1^. Thus, in control U251 cells, approximately 70% of the ATP was generated from glycolysis, with the remaining 30% generated from mitochondrial OXPHOS. In the copper-treated U251 cells, however, about 50% of the ATP was generated from mitochondrial OXPHOS (Figure 3A). Furthermore, exposure to copper increased the steady-state levels of intracellular ATP in the U251 and JX22 cells (Figure 3B). In agreement, cellular glucose content and lactate production were lower in the copper-treated cells than in the corresponding control cells (Figure 3C,D). These results indicate that bioavailable copper in GBM cells is associated with increased OXPHOS and decreased glycolysis.

**Figure 4:**
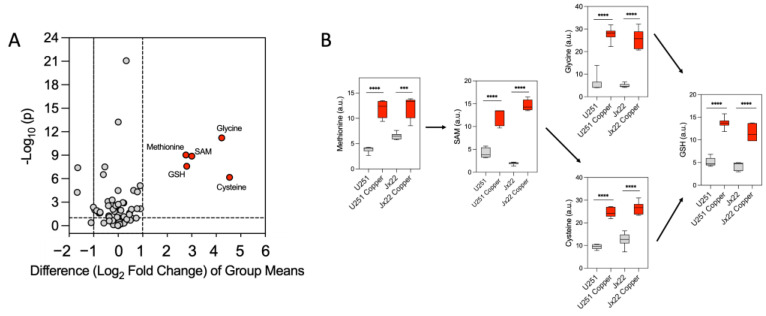
Excess copper upregulates glutathione metabolism in GBM cells. (**A**) Volcano plot of metabolomic data from cells treated with vehicle or 20 µM copper. The x-axis represents the mean fold change (log_2_ ratio) in the relative intensity of each metabolite between the two samples. The y-axis represents the statistical significance (−log_10_-transformed *p*-values) of each metabolite. (**B**) Boxplots depict the levels of key metabolites involved in the glutathione pathway analyzed in U251 and JX22 cells treated with vehicle or 20 µM copper for 48 h. Data are presented as the mean ± SEM (n = 6). *** *p* < 0.001 and **** *p* < 0.0001, calculated by Student’s *t* test.

**Figure 5:**
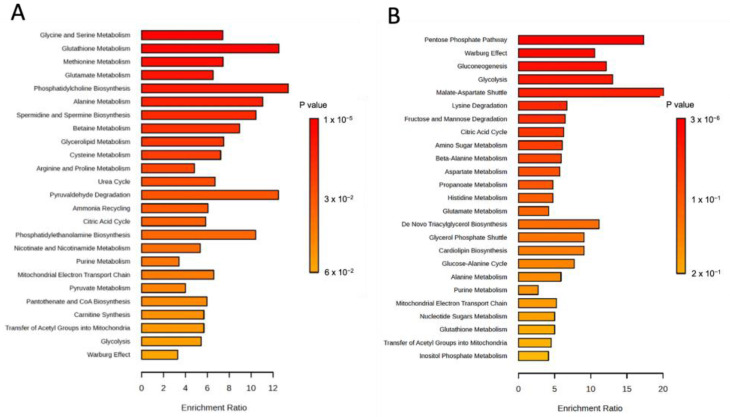
Pathway enrichment analysis of metabolites regulated by copper. This analysis was performed using significantly upregulated (**A**) and downregulated (**B**) metabolites identified in copper-treated cells. Enriched pathways were determined using the SMPDB database in MetaboAnalyst 6.0. Pathways are represented on the y-axis, ordered by significance (*p*-value), while the x-axis shows the pathway impact, reflecting the extent of metabolite involvement.

We then used targeted mass spectrometry-based metabolite profiling to examine relative levels of cell-associated metabolites in U251 and JX22 cells before and after 48 h of treatment with 20 µM copper (Supplementary Table S1). The most upregulated metabolites in copper-treated cells included reduced glutathione (GSH) and GSH biosynthesis intermediaries, including methionine, S-adenosyl methionine (SAM), cysteine, and glycine (Figure 4A,B). The sets of significantly altered metabolites were uploaded to MetaboAnalyst 6.0 for pathway enrichment analysis using the SMPBD database. The results, shown in Figure 5A,B, include statistically significant metabolites (*p* < 0.05). Figure 5A demonstrates that copper treatment highly enriched glycine, glutathione, methionine, and glutamate metabolism. In contrast, copper downregulated the pentose phosphate pathway, Warburg effect, and glycolysis (Figure 5B), indicating that copper treatment in GBM cells promotes increased OXPHOS and decreased glycolysis.

**Figure 6:**
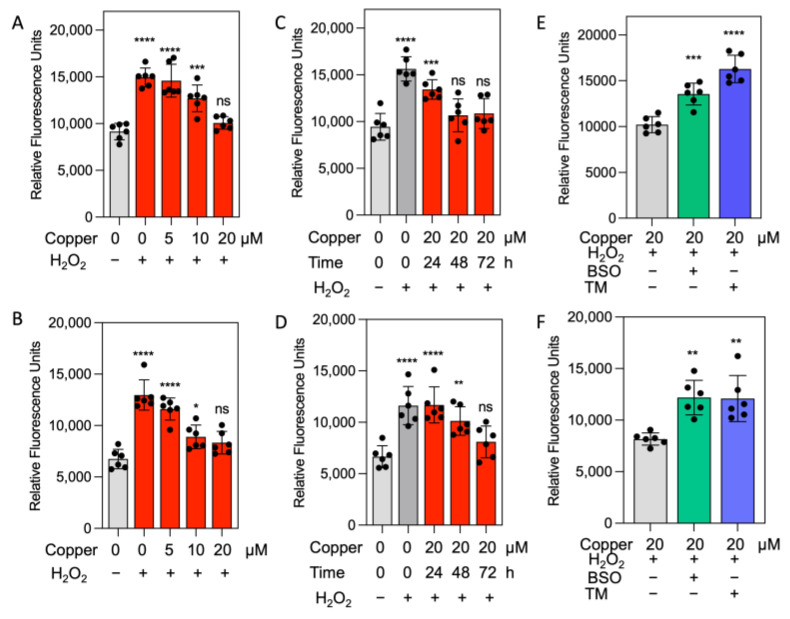
Dose–response and time-course analysis of copper’s effect on oxidative stress response to hydrogen peroxide. Dose–response effects of copper on H_2_O_2_-induced ROS in (**A**) U251 and (**B**) Jx22 glioma cells. Cells were treated with increasing concentrations of copper (0, 5, 10, and 20 µM) for 72 h before exposure to H_2_O_2_ for 30 min. Time-course analysis of ROS levels following treatment with 20 µM copper for 0, 24, 48, and 72 h, followed by 30 min of H_2_O_2_ exposure in (**C**) U251 and (**D**) Jx22 glioma cells. Effects of glutathione depletion (BSO) and copper chelation (TM) on copper-mediated protection against H_2_O_2_-induced oxidative stress, in (**E**) U251 and (**F**) Jx22 glioma cells. Relative fluorescence units reflect ROS levels, quantified using a ROS Detection Kit. Error bars represent mean ± SEM from triplicate determinations of two independent experiments. * *p* < 0.05 ** *p* < 0.01 *** *p* < 0.001, and **** *p* < 0.0001, calculated using one-way ANOVA followed by Tukey’s multiple comparison test. ns, no significant.

The observation that higher levels of glutathione and its precursors are present in copper-treated cells aligns with reduced oxidative stress. To further assess this, we evaluated the effect of copper on ROS generation in glioma cells using a Cellular Reactive Oxygen Species Detection Assay Kit. Treatment with 20 µM copper for 72 h significantly reduced H_2_O_2_-induced ROS production compared to vehicle control or lower copper doses (Figure 6A,B). At 5 µM copper, ROS levels induced by H_2_O_2_ were similar to vehicle control cells. A time-course analysis revealed a time-dependent reduction in ROS, with significant decreases after 48 h and non-significant levels after 72 h (Figure 6C,D). Furthermore, Figure 6E,F show that protection against H_2_O_2_-induced oxidative stress is both copper- and GSH-dependent, as it was reversed by treatment with BSO and TM. We did not observe any increase in oxidative stress in cells treated with copper compared to vehicle control cells. This suggests that the observed increase in metabolites involved in the glutathione pathway are upregulated by copper, contributing to protection against oxidative stress.

### 3.3. Bioavailable Copper Accelerates GBM Tumor Growth and Reduces Treatment-Induced GBM Cell Death

**Figure 7:**
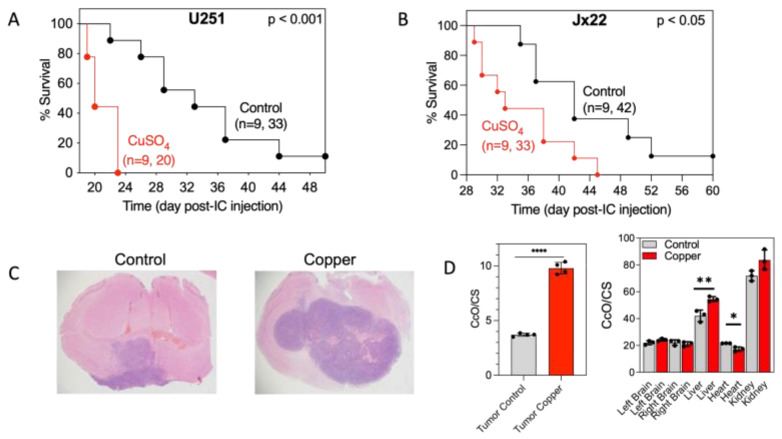
Excess copper promotes GBM tumor growth, increases tumor CcO activity, and decreases OS in vivo. (**A**,**B**) Kaplan–Meier survival curves of OS in nude mice harboring orthotopic brain tumors generated by intracranial inoculation with U251 (**A**) or JX22 (**B**) cells and administered Milli-Q water alone (controls) or Milli-Q water supplemented with 20 µM copper, with significance calculated by log-rank test. (**C**) Representative images of JX22 tumors harvested from control-treated and copper-treated mice and stained with hematoxylin and eosin. (**D**) CcO activity in tumor (left) or normal (right) tissue harvested from control-treated and copper-treated mice after euthanasia. CS, citrate synthase. Data are presented as the mean ± SEM. * *p* < 0.05, ** *p* < 0.01, and **** *p* < 0.0001, calculated by Student’s *t* test.

Because we previously demonstrated that high CcO activity is associated with increased tumorigenesis [42], we tested whether bioavailable copper administered in drinking water affects the survival of mice bearing intracranial GBM tumors. Among mice inoculated with U251 cells, overall survival (OS) was significantly lower for those in the copper-treated group than for those in the control group (20 days vs. 33 days, respectively). Similarly, among mice inoculated with JX22 cells, the OS was significantly lower for those in the copper-treated group than for those in the control group (33 days vs. 42 days, respectively) (Figure 7A,B). Notably, copper-treated mice also developed invasive tumors characterized by multifocal lesions throughout the brain parenchyma. In comparison, brains from control mice had only single lesions (Figure 7C). Overall, CcO activity was approximately 3-fold higher in tumor mitochondria from copper-treated mice than in tumor mitochondria from control mice. Interestingly, no significant copper-related differences in CcO activity were detected in mitochondria isolated from most other organs, with the exception of liver and heart in which CcO activity was slightly higher and lower, respectively, in copper-treated mice (Figure 7D). These results are in agreement with the high rate of copper uptake by cancer cells compared with normal cells (Figure 1E). Overall, these results suggest that copper promotes GBM tumor growth and aggression.

**Figure 8:**
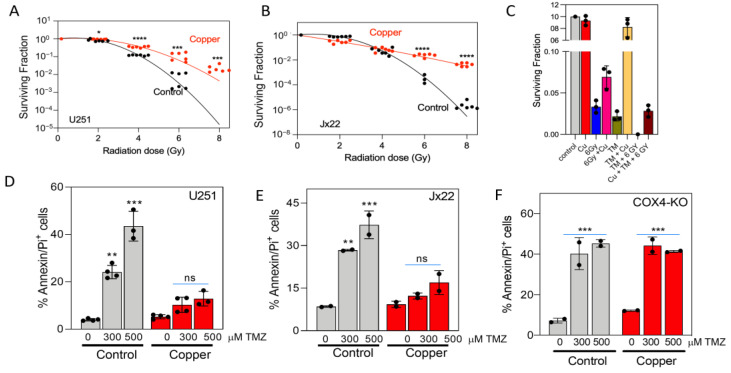
Excess copper increases resistance to radiation and TMZ. (**A**,**B**) Clonogenic survival curves for U251 (**A**) and JX22 (**B**) cells treated with vehicle or 20 µM copper. Cells were irradiated with 2, 4, 6, or 8 Gy and immediately plated. Clonogenic survival was estimated on day 14 after irradiation. (**C**) Clonogenic survival for U251 cells treated with vehicle or 20 µM copper alone or in combination with 10 µM TM. Cells were irradiated with 6 Gy and immediately plated. Clonogenic survival was estimated on day 14 after irradiation. (**D**,**E**) Flow cytometry analysis with annexin V-PI staining in U251 (**D**) and JX22 (**E**) cells treated with vehicle or 20 µM copper alone or in combination with TMZ at the doses shown. (**F**) Flow cytometry analysis with annexin V-PI staining in U251-deltaCOX4 cells treated with vehicle or 20 µM copper alone or in combination with TMZ at the doses shown. (**A**–**F**) * *p* < 0.05, ** *p* < 0.01, and *** *p* < 0.001, **** *p* < 0.0001, calculated using one-way ANOVA followed by Tukey’s multiple comparison test. ns, not significant. (**E**,**F**) Data are presented as the mean ± SEM.

We next characterized the status of the in vitro tumor cell response to radiation exposure in the presence or absence of copper using clonogenic survival assays. Exposure to copper significantly increased the resistance to radiation-induced cell death in U251 and JX22 cells (Figure 8A,B). As shown in Figure 8C, co-treatment with the copper chelator TM abrogated the copper-induced increase in radioresistance (Figure 8C). Using annexin V/PI staining and flow cytometry, we also examined the effects of copper on the induction of GBM cell apoptosis by the chemotherapeutic agent TMZ. Treatment with 300 μM and 500 μM TMZ for 48 h caused a 25% and 45% increase, respectively, in the number of annexin V/PI-positive cells in U251 control cells, but exposure to copper prevented this apoptotic effect. Copper exposure similarly protected JX22 cells from TMZ-induced apoptosis (Figure 8D,E). However, copper exposure did not protect COX4-depleted cells from TMZ-induced apoptosis, suggesting the direct involvement of CcO in the copper-mediated chemoresistance (Figure 8F).

## 4. Discussion

The relationship between copper and tumors was first established in 1965 when de Jorge et al. reported an 11-fold increase in copper levels in brain cancer, marking the initial connection between elevated copper levels and tumors [50,51]. Subsequent studies, including Turecky et al. [52], confirmed that serum copper levels in brain tumor patients are significantly higher compared to healthy individuals or those with non-tumorous neurological diseases. Copper levels also correlated with disease progression, being highest in patients with advancing disease. Additionally, malignant glioma tissues were found to have significantly higher copper levels than normal brain tissues [53].

Elevated copper levels have been associated with the growth and metastasis of certain types of cancer [34,35,36], but whether copper has similar effects in GBM has remained unknown. The results of this study confirm that increasing bioavailable copper levels not only promote GBM cell proliferation but also enhance treatment resistance in GBM cells by upregulating mitochondrial CcO activity within the cells. Furthermore, our results indicate that the long-term ingestion of copper at the upper limit allowed in public drinking water by the EPA, 20 µM, exacerbates GBM tumor growth and invasion in vivo.

In the patient-derived GBM cell lines used in this study, in vitro exposure to copper increased CcO activity in a dose- and time-dependent manner, with significant increases in activity observed at copper concentrations as low as 5 µM. Confirming the specificity of these results, the copper-induced activation of CcO was not detected in COX4-depleted GBM cells that are unable to assemble the CcO holoenzyme or in cells simultaneously exposed to the copper chelator TM, and the activity of SOD, another copper-dependent enzyme, was not affected. A further analysis of ATP production, lactate production, and glucose uptake revealed that exposure to 20 µM copper was sufficient to induce an OXPHOS-dominated metabolic phenotype in these cells. In mice with orthotopic GBM tumors, consumption of drinking water supplemented with 20 µM copper exacerbated tumor growth and invasion and reduced overall survival.

Copper-dependent enzymes such as CcO—a key enzyme in OXPHOS—are essential in ATP production, which is often upregulated in cancer cells. Furthermore, OXPHOS is increasingly recognized as a key factor in therapy resistance across multiple cancer types, including GBM [41,44,54,55,56]. While cancer cells were once believed to rely predominantly on glycolysis even in oxygen-rich environments (the Warburg effect), recent studies reveal that many tumors—particularly those resistant to chemotherapeutic drugs—can flexibly switch to or maintain high OXPHOS levels. This metabolic adaptation provides a survival advantage, as OXPHOS-generated ROS can activate growth and survival signaling pathways. Although ours is the first study to report the tumor-promoting effects of excess copper in GBM, our finding that these effects are mediated through the specific activation of CcO is not without precedence. Ishida et al. previously reported that the long-term consumption of 20 µM copper in drinking water stimulated CcO activity in tumor cells as well as tumor cell proliferation and de novo pancreatic tumor growth in RIP1-Tag2 mice. Oral administration of TM, however, was associated with lower CcO activity and ATP levels in the tumors and diminished tumor growth. In cells derived from the RIP1-Tag2 tumors, the anti-proliferative effects of copper chelation were further enhanced by glycolysis inhibitors. These findings, along with the results of our study, suggest that the upregulation of glycolysis in tumors may partially reflect insufficient copper bioavailability within the tumor microenvironment [34].

Research by Ramchandani et al. also revealed elevated intracellular copper levels and pronounced sensitivity to TM in a subpopulation of highly metastatic SOX2/OCT4+ cells within primary triple-negative breast cancer (TNBC) tumors [36]. Additional global proteomic and metabolomic analyses identified that treatment with TM reduced the abundance and activity of CcO in these cells, which significantly reduced OXPHOS-mediated ATP production. In contrast to our results, showing that copper exposure enhances GBM cell proliferation, tumor invasion, and resistance to treatment, however, their results indicated that copper only regulated the invasiveness of TNBC tumors, likely through the regulation of AMPK [36]. Therefore, the tumor-promoting effects of copper-induced CcO activity may differ to some extent depending on the tumor cell type.

An increased reliance on OXPHOS can also increase ROS production in cancer cells, which in turn stimulates antioxidant defenses, including GSH, allowing the cells to harness the benefits of ROS-driven signaling while avoiding toxic oxidative damage. High levels of GSH have been implicated in tumor initiation and proliferation [57] and may in part explain the enhanced GBM tumor growth and invasion we observed in mice exposed to copper. GSH has also been shown to promote treatment resistance [58] in cancer cells, including brain cancer cells [59,60,61]. Indeed, our previous work revealed that ROS production is attenuated in U251 cells with acquired resistance to TMZ, and treatment with the GSH inhibitor buthionine sulfoximine (BSO) restored TMZ-induced ROS production and apoptosis in these cells [43]. Additionally, Rocha et al. detected high levels of GSH in chemoresistant U138MG cells and showed that treatment with BSO restored the cell sensitivity to cisplatin and TMZ [62]. The OXPHOS-mediated upregulation of GSH we observed in copper-treated GBM cells may similarly explain the resistance of these cells to radiation and TMZ. Supporting this hypothesis, deltaCOX4 GBM cells in which CcO cannot be activated remained sensitive to radiation and TMZ after treatment with copper.

In fact, the upregulation of GSH may also explain the lack of cuproptosis in these cells. A study by Scheiber and Dringen [63] showed that the accumulation of GSH protects NHAs from copper-induced toxicity, as exposure of astrocyte-rich primary cultures to copper chloride for 24 h led to substantial copper accumulation and increased cellular GSH content by up to 165% without affecting GSH disulfide (GSSG) levels. The protective effect of GSH appears to hold true in cancer cells, too. In particular, a study with copper-resistant hepatoma cells demonstrated that elevated GSH levels, along with metallothioneins and GSH peroxidase, supported cellular resistance to copper toxicity by sequestering free cytoplasmic copper as well as by mitigating the oxidative damage induced by the copper [64]. Indeed, the treatment of A549 lung cancer cells with therapeutic copper-leaching nanoparticles was recently shown to induce a compensatory response that included the upregulation of GSH and NRF2-activated antioxidant pathways, among other responses that limit the copper-induced toxicity [65].

Copper is essential for CcO function, and our findings suggest that many GBM cells—particularly therapy-resistant GBM cells—rely heavily on copper-dependent CcO activity to sustain OXPHOS. Although treatment was not the focus of our study, this information further supports GSH as a target for adjuvant therapies designed to resensitize GBM cells to radiation and chemotherapy. A few published studies have already pointed to the validity of such strategies in GBM, including our study [43] and the study by Rocha et al. [62] that showed that treatment with BSO restores sensitivity to treatment in glioma cells. Copper chelators, which reduce the amount of bioavailable copper, may also offer a promising therapeutic approach for GBM by targeting the role of copper in the OXPHOS-driven survival mechanisms we have detected (e.g., cellular proliferation and treatment resistance), as well as by targeting copper-driven angiogenesis [32,33,66,67,68,69,70].

## 5. Conclusions

In conclusion, our work identifies copper as a GBM tumor promoter by demonstrating that long-term exposure to elevated copper levels in drinking water—within the maximum limits allowed in public water supplies—accelerates tumor growth in mice. Our findings also reveal a role for copper in regulating CcO activity in GBM cells, contributing to resistance against radio- and chemotherapy. These insights enhance our understanding of the metabolic adaptations in cancer cells and highlight copper-dependent pathways as promising therapeutic targets.

## Data Availability

The original contributions presented in this study are included in the article/Supplementary Materials.

## Supplementary Materials

The following supporting information can be downloaded at: https://www.mdpi.com/article/10.3390/antiox14020142/s1.
